# Analysis of a Modified Version of the Inventory of Non‐Ataxia Signs Over 12 Years in Patients with Friedreich's Ataxia in the EFACTS Study

**DOI:** 10.1002/mds.70084

**Published:** 2025-10-10

**Authors:** Stella Andrea Lischewski, Imis Dogan, Paola Giunti, Michael H. Parkinson, Caterina Mariotti, Alexandra Durr, Claire Ewenczyk, Sylvia Boesch, Wolfgang Nachbauer, Thomas Klopstock, Claudia Stendel, Francisco Javier Rodríguez de Rivera Garrido, Ludger Schöls, Zofia Fleszar, Thomas Klockgether, Marcus Grobe‐Einsler, Ilaria Giordano, Myriam Rai, Massimo Pandolfo, Heike Jacobi, Ralf‐Dieter Hilgers, Jörg B. Schulz, Kathrin Reetz, the EFACTS Study Group, Elisabetta Indelicato, Matthias Amprosi, Cinzia Gellera, Alessia Mongelli, Anna Castaldo, Mario Fichera, Enrico Bertini, Gessica Vasco, Marie Biet, Marie Lorraine Monin, Florian Holtbernd, Nikolina Brcina, Christian Hohenfeld, Florentine Radelfahr, Almut T. Bischoff, Stefanie N. Hayer, Georgios Koutsis, Marianthi Breza, Francesc Palau, Mar O'Callaghan, Gilbert Thomas‐Black, Katarina Manso, Nita Solanky, Robyn Labrum

**Affiliations:** ^1^ Department of Neurology RWTH Aachen University Aachen Germany; ^2^ JARA‐BRAIN Institute Molecular Neuroscience and Neuroimaging, Forschungszentrum Jülich GmbH Jülich Germany; ^3^ Ataxia Centre, Department of Clinical and Movement Neurosciences UCL‐Queen Square Institute of Neurology London UK; ^4^ Unit of Medical Genetics and Neurogenetics, Fondazione IRCCS Istituto Neurologico Carlo Besta Milan Italy; ^5^ Sorbonne Universite, Paris Brain Institute, ICM Institut du Cerveau, AP‐HP, INSERM, CNRS, University Hospital Pitié‐Salpêtrière Paris France; ^6^ Department of Neurology Medical University Innsbruck Innsbruck Austria; ^7^ Friedrich‐Baur‐Institute, Department of Neurology, LMU University Hospital Ludwig‐Maximilians‐Universität München Munich Germany; ^8^ German Center for Neurodegenerative Diseases (DZNE) Munich Germany; ^9^ Munic Cluster for Systems Neurology (SynNergy) Munich Germany; ^10^ Reference Unit of Hereditary Ataxias and Paraplegias, Department of Neurology, IdiPAZ, Hospital Universitario La Paz Madrid Spain; ^11^ Department of Neurology and Hertie‐Institute for Clinical Brain Research University of Tübingen Tübingen Germany; ^12^ German Center for Neurodegenerative Diseases Tübingen Germany; ^13^ German Center for Neurodegenerative Diseases Bonn Germany; ^14^ Department of Neurology University Hospital of Bonn Bonn Germany; ^15^ Friedreich's Ataxia Research Alliance Pennsylvania USA; ^16^ Laboratory of Experimental Neurology, Université Libre de Bruxelles Brussels Belgium; ^17^ Department of Neurology University of Heidelberg Heidelberg Germany; ^18^ Department of Medical Statistics RWTH Aachen University Aachen Germany

**Keywords:** Friedreich's ataxia, longitudinal, non‐ataxia symptoms

## Abstract

**Background:**

Friedreich's ataxia is a rare, neurodegenerative, multisystem disorder. While ataxia is a hallmark, non‐ataxia signs, including muscle weakness, spasticity, and dysphagia are equally disabling. The Inventory of Non‐Ataxia Signs (INAS) is a symptom list transformable to a 16‐item count.

**Objective:**

To evaluate the responsiveness of a modified INAS in this population.

**Methods:**

Participants were drawn from the European Friedreich's Ataxia Consortium for Translational Studies (EFACTS). The modified INAS count (presence/absence, 0–16 scale) and modified INAS sum (severity‐weighted, 0–84 scale) were evaluated using linear mixed‐models and standardized response means (SRMs). Items rare (<5%) and uncharacteristic in Friedreich's ataxia were excluded (chorea, myoclonus, fasciculations, resting tremor, rigidity)

**Results:**

A total of 1129 participants (mean age, 32.3 years) were assessed for up to 12 years. The mean modified INAS count was 4.6 (±2.2) and modified INAS sum 15.1 (± 9.9). Both correlated strongly with existing outcome measures. Longitudinally, the modified INAS count increased by 0.13 points/year (95% CI 0.12, 0.14; *P* < 0.001) and modified INAS sum by 0.68 points/year (95% CI 0.64, 0.72; *P* < 0.001). The modified INAS sum demonstrated greater responsiveness, with SRMs of 0.26, 0.38, 0.53, and 0.80 at 1, 2, 3, and 5 years, respectively, compared with 0.16, 0.27, 0.31, and 0.46 for the modified INAS count. In non‐ambulatory patients and children, responsiveness of the modified INAS sum was higher (SRM 0.82 and 1.7 at 5 years, respectively).

**Conclusions:**

The modified INAS sum showed good responsiveness over 5 years but not over 1–3 years. It may supplement existing outcome measures, contributing to holistic assessment of this multisystem disease, especially in non‐ambulatory patients, in whom ataxia‐focused measures may show ceiling effects, and children, who typically progress faster. © 2025 The Author(s). *Movement Disorders* published by Wiley Periodicals LLC on behalf of International Parkinson and Movement Disorder Society.

## Introduction

Friedreich's ataxia is a progressive, multisystem, neurodegenerative disorder inherited in an autosomal recessive pattern. The majority of individuals with Friedreich's ataxia harbor biallelic GAA repeat expansions in the *FXN* gene, leading to a deficiency of the mitochondrial protein frataxin. While larger GAA expansions are known to confer a prognostic disadvantage, the highly heterogenous phenotype of Friedreich's ataxia remains insufficiently explained by genetic factors, which has presented a major barrier to the development of treatments.^1^, ^2^, ^3^ In addition to ataxia, individuals with Friedreich's ataxia exhibit a spectrum of neurological symptoms, including spasticity, muscle weakness, bladder dysfunction, and dysphagia, as well as non‐neurological manifestations such as scoliosis, foot deformities, cardiomyopathy, and diabetes.^4^ These non‐ataxia symptoms substantially impair daily living activities, may increase dependence on caregivers, and adversely affect quality of life.

With the first medication, omaveloxolone, recently approved for Friedreich's ataxia^5^, ^6^ and further potential therapies on the horizon, validated, reliable, and responsive outcome measures are essential for evaluating therapeutic efficacy. Current outcome measures include the Scale for the Assessment and Rating of Ataxia (SARA)^7^ and the modified Friedreich's Ataxia Rating Scale (mFARS),^8^, ^9^ which focus on ataxia progression, as well as the Activities of Daily Living (ADL) scale, which is a component of the original Friedreich's Ataxia Rating Scale (FARS) and assesses functional impairment.^10^, ^11^ To our knowledge, non‐ataxia symptoms have not been incorporated into outcome assessments in present clinical trials in Friedreich's ataxia; however, their inclusion may improve content validity, ensuring a broader range of disabling symptoms are considered. This is particularly the case for non‐ambulatory individuals, in whom non‐ataxia symptoms appear to be more prevalent and ataxia‐based measures may be limited by ceiling effects.^12^

The Inventory of Non‐Ataxia Signs (INAS) is a 30‐item symptom list assessing non‐cerebellar involvement that can be transformed into a semi‐quantitative 16‐item count.^13^ The INAS is clinician‐administered, designed to align with the routine neurological examination, requires no specific training, and can be completed in approximately 10 minutes. The INAS count evaluates the presence or absence of neurological non‐ataxia signs and symptoms (areflexia, hyperreflexia, extensor plantar response, spasticity, paresis, muscle atrophy, fasciculations, myoclonus, rigidity, chorea, dystonia, resting tremor, sensory symptoms, brainstem oculomotor signs, urinary dysfunction, and cognitive impairment). It thus indicates the quantity but not the severity of non‐ataxia symptoms, a higher score corresponds to worse impairment. It has been devised in patients with spinocerebellar ataxia, demonstrating high inter‐rater and short‐term test–retest reliability but poor responsiveness over a 2‐year follow‐up period.^13^

Nonetheless, in view of the higher burden of non‐ataxia symptoms in patients with Friedreich's ataxia compared with spinocerebellar ataxias, we hypothesized that the INAS has potential as an outcome measure in patients with Friedreich's ataxia, particularly in non‐ambulatory patients. We believe it has the potential to yield valuable information from the routine neurological examination to quantify progression, particularly in later disease stages. Our objectives were therefore to investigate the longitudinal evolution of non‐ataxia symptoms and to assess the responsiveness of a modified INAS count in this patient population over a follow‐up period of 12 years. In addition to the modified INAS count, we sought to introduce a modified INAS sum, which accounts for the severity of non‐ataxia symptoms. Our results were compared with previous work on the same study cohort focusing on progression characteristics, particularly the SARA and ADL.^1^, ^14^, ^15^

## Methods

### Participants and Assessments

Participants were drawn from the European Friedreich's Ataxia Consortium for Translational Studies (EFACTS) registry. Detailed methods have been described previously.^1^, ^14^, ^15^ Written informed consent was acquired prior to participation. Patients with genetically confirmed Friedreich's ataxia were assessed annually in 15 centers across Europe. Ataxia progression was measured using the SARA (0–40 scale), while impairment in activities of daily living was evaluated using the ADL scale (0–36 scale), with higher scores indicating greater impairment.

The INAS is a 30‐item clinical instrument and symptom list assessing neurological non‐cerebellar involvement and can be transformed into the INAS count, which considers the presence or absence of 16 neurological signs and symptoms. Items are rated as absent, mild, moderate, or severe and are denoted scores of 0, 1, 2, or 3, respectively. Exceptions include brainstem oculomotor signs, which are binary, and reflexes, which are categorized as present, absent, and hyperreflexia.^13^

To adapt the original INAS for use in Friedreich's ataxia, we introduced several modifications aimed at improving longitudinal responsiveness and clinical relevance (Table S1). These were based on empirical data from our study cohort and a review of published data on non‐ataxia symptom prevalence in Friedreich's ataxia (Table S2). Items were excluded if they were observed in fewer than 5% of patients and lacked clinical relevance. Conversely, symptoms listed in the INAS reported symptoms but not originally included in the INAS count – such as dysphagia and muscle cramps – were added due to their frequency and relevance in this population. Consequently, we excluded chorea, fasciculations, myoclonus, resting tremor, and rigidity. Although dystonia was observed in 4.5% of patients, it was retained as this was considered sufficiently close to the 5% threshold. Furthermore, dystonia was deemed pathophysiologically plausible in Friedreich's ataxia, particularly in the context of spastic dystonia in advanced disease stages^16^ and the role of axial dystonia or spinal spasms in development of scoliosis.^17^ We performed sensitivity analysis and exploratory factor analysis of the original INAS to test the validity of these modifications. In addition to the modified INAS count, we calculated a modified INAS sum by summing the severity of individual items, yielding a total score ranging from 0 to 84. We also developed the modified INAS responsive sum, which was developed from the modified INAS sum (ie, underwent the same modifications) but only incorporates items with good internal responsiveness. Patients with onset above 25 years of age were considered to have a late onset and those with onset before and including 25 years to have typical onset.

### Statistical Analysis

Correlations were assessed using Pearson's correlation coefficient. Associations between baseline clinical and demographic factors and the modified INAS were evaluated via univariate and multivariable linear regression. Variance inflation factors were examined to detect multicollinearity, and scatter plots were used to assess outliers and linearity. Q‐Q plots were reviewed to confirm the normality of residuals. Given the exploratory nature of the regression analysis, correction for multiple comparison was not performed.

The longitudinal evolution of the modified INAS was analyzed using linear mixed‐effects models with a random intercept and fixed slope due to difficulties with model convergence when fitting a random slope. Although models with higher‐order polynomials were tested, the linear model provided the best fit. Interactions between follow‐up time and ambulatory status were included in the analysis. Binary modified INAS count items were analyzed using time‐to‐event methods, with Kaplan–Meier curves illustrating the time from baseline to symptom onset.

Sample sizes for hypothetical clinical trials of the modified INAS count and modified INAS sum were estimated based on observed progression rates in linear mixed‐effects models. Calculations assumed 80% power, a type‐1 error rate of 0.05, 1:1 allocation, and a treatment benefit equivalent to three times the annual progression rate.

Internal responsiveness, defined as the ability of the modified INAS to detect change over time, was assessed using standardized response means (SRMs). The SRM was calculated by dividing the mean change by the standard deviation of the change,^18^ with values <0.5 indicating low responsiveness, 0.5–0.8 moderate responsiveness, and >0.8 high responsiveness.^19^ External responsiveness, defined as the relationship between INAS changes and reference measures,^18^ was evaluated using Pearson correlation coefficients and linear mixed‐effects models, with the SARA and ADL scales serving as reference measures.

Exploratory factor analysis was conducted to assess the underlying factor structure of the modified and original INAS at baseline. Bartlett's test of sphericity tested the suitability for factor analysis, and the Kaiser–Meyer–Olkin test assessed sampling adequacy. The number of factors was decided upon visual inspection of a scree plot and considering the substantive interpretability. Items with loadings ≥0.25 were retained for interpretation, and communalities (h^2^) were used to assess the proportion of variance explained by the factors. Item‐to‐item and item‐total correlations were also examined.

All *P*‐values were two‐tailed, with significance set at *P* < 0.05. Descriptive statistics are reported as mean ± standard deviation (SD) or median and interquartile range (IQR). Analyses were conducted in Python 3.12.0 (using libraries such as *statsmodels*, *sksurv*, and *lifelines*) and R 4.4.1 (utilizing *gtsummary*, *TrialSize*, *psych*, and others).

## Results

### Baseline Characteristics

A total of 1129 participants (962 adults and 167 children) made 5567 visits, with a median of three visits per participant. At baseline, the mean modified INAS count (0–16 scale) was 4.6 (± 2.2) and the mean modified INAS sum (0–84 scale) was 15.1 (± 9.9). The most common symptoms and signs reported in 54%–88% of participants were areflexia, impaired vibration sensation, positive extensor plantar response, dysphagia, and muscle weakness, while hyperreflexia and dystonia were uncommon, occurring in 5%–7%. All symptoms, except hyperreflexia, were more frequent in non‐ambulatory patients compared with ambulatory patients (Table 1).

**TABLE 1:** Main demographic and clinical characteristics of study participants at baseline

Characteristic	Overall	Non‐ambulatory	Ambulatory
	(N = 1129)	(N = 487)	(N = 642)
Age, years	30 (21, 42)	33 (25, 44)	26 (19, 41)
Adult	962 (85%)	469 (96%)	493 (77%)
Sex, female	571 (51%)	249 (51%)	322 (50%)
Age at symptom onset, years	13 (8, 19)	11 (8, 15)	15 (10, 24)
Disease duration, years	15 (9, 23)	22 (16, 30)	10 (6, 14)
GAA repeats shorter allele	600 (367, 767)	700 (567, 834)	500 (250, 683)
GAA repeats longer allele	900 (750, 1000)	912 (834, 1020)	850 (680, 1000)
SARA score	19 (10, 29)	30 (26, 33)	11 (9, 15)
Cardiac hypertrophy	391 (41%)	205 (49%)	186 (34%)
Modified INAS sum	12 (8, 20)	22 (17, 28)	9 (6, 11)
Modified INAS count	5 (3, 6)	6 (5, 7)	3 (2, 4)
Spasticity	306 (28%)	208 (44%)	98 (16%)
Muscle weakness	587 (54%)	427 (91%)	160 (26%)
Muscle atrophy	470 (43%)	346 (73%)	124 (20%)
Dystonia	50 (4.5%)	36 (7.6%)	14 (2.2%)
Impaired vibration sensation	924 (87%)	436 (96%)	488 (80%)
Urinary dysfunction	406 (37%)	263 (55%)	143 (23%)
Dysphagia	628 (57%)	390 (82%)	238 (38%)
Areflexia	970 (88%)	462 (97%)	508 (81%)
Hyperreflexia	78 (7.1%)	15 (3.2%)	63 (10%)
Extensor plantar reflex	699 (64%)	358 (76%)	341 (55%)
Oculomotor dysfunction	300 (29%)	201 (46%)	99 (17%)
Cognitive impairment	62 (5.6%)	45 (9.5%)	17 (2.7%)
Muscle cramps	476 (43%)	266 (56%)	210 (34%)

*Note*: Values are either number (percentage) or median (range).

Abbreviations: SARA, Scale for the Assessment and Rating of Ataxia; INAS, Inventory of Non‐Ataxia Signs.

Disease duration, GAA repeat length on the shorter allele, ADL and SARA scales, and presence of cardiac hypertrophy were significantly associated with both modified INAS scores in univariate regression and age of onset was inversely associated (Table S3).

### Availability of Follow‐up Data for the Modified INAS

Missing data were highest in the subitem gait spasticity, which was missing in approximately 17%–39% of patients depending on the visit. The high missingness in this item may have arisen due to confusion over how to rate this item in non‐ambulatory patients. Missingness for other items was low (<10% in most visits). It was therefore decided to exclude gait spasticity from both the modified INAS count and modified INAS sum. Doing so yielded missingness of 16% at visit 1, 13% at visit 3, 24% at visit 5, 37% at visit 10, and 17% at visit 12 in the modified INAS count and modified INAS sum. The number of patients followed up for each visit can be found in Table S4 and the missingness for individual items in Tables S5 and S6. Participants with missing modified INAS count data had slightly higher mean SARA and ADL scales than those with available scores.

### Internal Responsiveness and Longitudinal Evolution of the Modified INAS

The modified INAS sum exhibited substantially higher responsiveness across all intervals compared with the modified INAS count. The modified INAS sum showed poor to moderate internal responsiveness after 1–3 years follow‐up but high internal responsiveness after 5 years follow‐up, while the modified INAS count showed poor internal responsiveness after 5 years follow‐up and moderate responsiveness after 10 years (Table 2). Sensitivity analysis of the INAS sum, including the previously excluded items fasciculations, chorea, rigidity, myoclonus, and resting tremor, yielded an INAS sum score with slightly worse performance compared with the modified INAS sum with SRMs of 0.25, 0.36, 0.49, 0.77, and 1.07, respectively, for 1, 2, 3, 5, and 10 years of follow‐up.

**TABLE 2:** Standard response means of the modified Inventory of Non‐Ataxia Signs (INAS) sum subitems

Cohort	Item	1‐year follow‐up (n = 832)	2‐year follow‐up (n = 732)	3‐year follow‐up (n = 544)	5‐year follow‐up (n = 454)	10‐year follow‐up (n = 157)
Total cohort (N = 1129 at baseline)	Modified INAS sum	0.26	0.38	0.53	0.80	1.08
	Modified INAS count	0.16	0.27	0.31	0.46	0.63
	Modified INAS responsive sum	0.30	0.42	0.48	0.72	1.09
	Areflexia	−0.01	−0.01	−0.02	0.04	0.08
	Hyperreflexia	0.02	0.01	−0.03	0.05	0.07
	Extensor plantar reflex	0.05	0.13	0.12	0.13	0.13
	Spasticity	0	−0.04	0.07	0.10	0.27
	Muscle weakness	0.2	0–28	0.33	0.54	0.82
	Muscle atrophy	0.11	0.18	0.27	0.44	0.67
	Dystonia	0.01	0.10	0.05	0.15	0.28
	Impaired vibration sensation	0.20	0.27	0.40	0.53	0.83
	Oculomotor dysfunction	0.10	0.07	0.20	0.07	0.09
	Urinary dysfunction	0.09	0.18	0.16	0.32	0.56
	Cognitive impairment	0	−0.01	0.07	0.09	0.19
	Dysphagia	0.04	0.15	0.13	0.30	0.40
	Muscle cramps	0.13	0.08	0.12	0.19	0.31
Ambulatory patients (n = 642)	Modified INAS sum	0.32	0.53	0.64	0.75	0.99
	Modified INAS count	0.25	0.42	0.54	0.60	0.76
	Modified INAS responsive sum	0.29	0.47	0.44	0.68	1.03
Non‐ambulatory patients (n = 487)	Modified INAS sum	0.20	0.26	0.47	0.82	1.02
	Modified INAS count	−0.01	0.01	−0.01	0.11	0.23
	Modified INAS responsive sum	0.31	0.30	0.50	0.69	0.97
Typical‐onset patients (n = 974)	Modified INAS sum	0.24	0.35	0.57	0.85	1.16
	Modified INAS count	0.12	0.21	0.28	0.41	0.55
	Modified INAS responsive sum	0.30	0.43	0.54	0.78	1.18
Late‐onset patients (n = 155)	Modified INAS sum	0.37	0.58	0.37	0.64	1.31
	Modified INAS count	0.40	0.62	0.43	0.63	0.96
	Modified INAS responsive sum	0.33	0.44	0.24	0.56	1.21
Adults (n = 962)	Modified INAS sum	0.24	0.35	0.47	0.72	0.99
	Modified INAS count	0.11	0.23	0.24	0.37	0.50
	Modified INAS responsive sum	0.19	0.31	0.43	0.69	1.01
Children (n = 162)	Modified INAS sum	0.45	0.68	1.07	1.71	NA
	Modified INAS count	0.43	0.54	0.77	1.64	NA
	Modified INAS responsive sum	0.46	0.66	0.91	1.49	NA

Abbreviations: INAS, Inventory of Non‐Ataxia Signs; NA, not available.

The subitems with consistent responsiveness over time included muscle weakness, muscle atrophy, impaired vibration sensation, urinary dysfunction, and dysphagia. Spasticity and dystonia showed responsiveness only at later intervals but not in the short term and the responsiveness of dystonia was slightly inconsistent between time points. In contrast, extensor plantar reflex, hyperreflexia, areflexia, and oculomotor dysfunction demonstrated poor responsiveness over the study duration. To improve sensitivity, the modified INAS responsive sum (0–48 scale, mean 9.0 ± 8.2) was developed, incorporating only items with consistent responsiveness, which included muscle weakness, muscle atrophy, impaired vibration sensation, urinary dysfunction, dysphagia, and spasticity. However, it showed superior responsiveness only at 1‐ and 2‐year follow‐up but not at 3‐, 5‐, or 10‐year follow‐up (Table 2).

The modified INAS count increased significantly by 0.13 points/year (standard error [SE] ± 0.007 [95% CI 0.12, 0.14]; *P* < 0.001), while the modified INAS sum increased significantly by 0.68 points/year (SE ± 0.02 [95% CI 0.64, 0.72]; *P* < 0.001). A significant interaction between visit and ambulatory status was observed for the modified INAS count but not the modified INAS sum, indicating that the increase in the modified INAS count was 0.19 points less in non‐ambulatory patients compared with ambulatory patients.

**FIG. 1:**
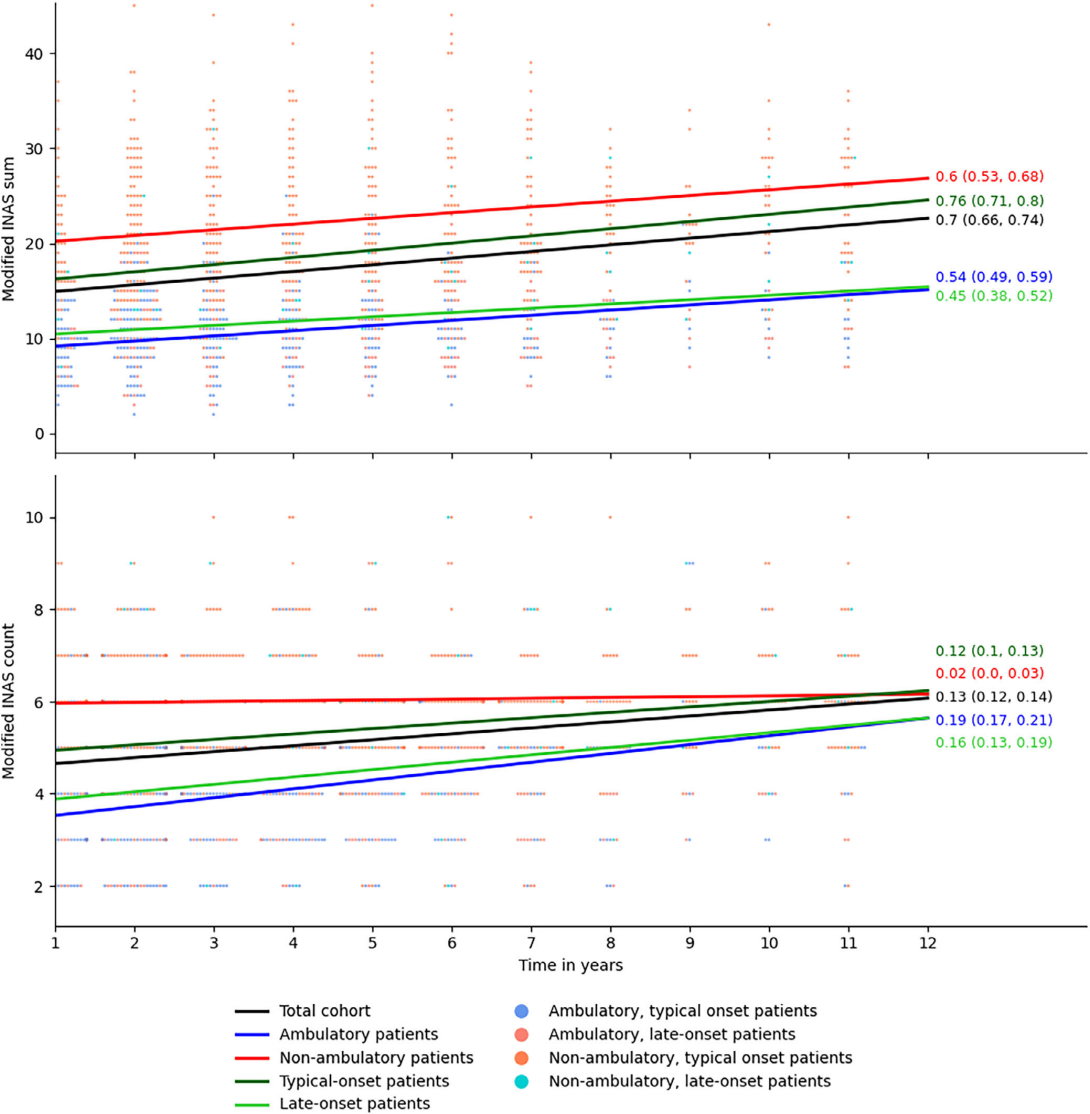
Longitudinal evolution of the modified Inventory of Non‐Ataxia Signs (INAS) summed and the modified INAS count total score. Results of linear mixed‐effects models for the modified INAS sum total score (upper graph) and modified INAS count (lower graph) in different subcohorts. The annotations denote the linear regression coefficient with confidence intervals. The scatter points are drawn from a random sample of the dataset (n = 400) and represent individual participant scores. [Color figure can be viewed at wileyonlinelibrary.com]

**FIG. 2:**
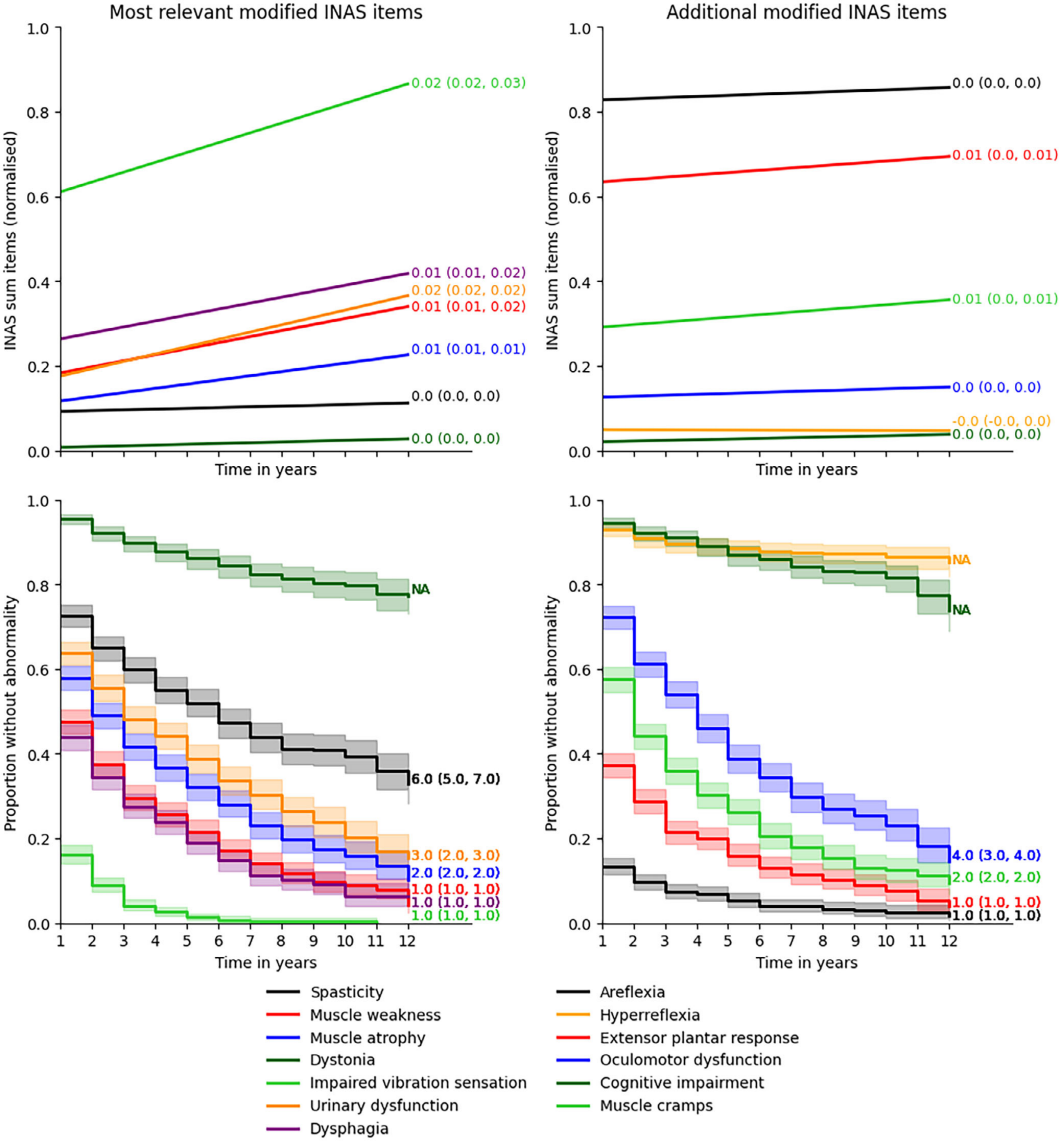
Longitudinal evolution of modified Inventory of Non‐Ataxia Signs (INAS) subitems over time. The upper graph shows the results of linear mixed effects models for the normalized (0–1 scale) modified INAS summed items. The annotations denote the linear regression coefficient with confidence intervals. The lower graph shows the Kaplan–Meier curves for the modified INAS count items (present/absent scale). The annotations denote the median time until symptom onset with confidence intervals. [Color figure can be viewed at wileyonlinelibrary.com]

Analysis of subcohorts revealed the fastest progression of the modified INAS sum and modified INAS count in children. The slowest progression of the modified INAS sum occurred in ambulatory patients and those with late‐onset disease and of the INAS count in non‐ambulatory patients, who showed almost no progression (Fig. 1). Median time until symptom onset was relatively short (1–2 years) for impaired vibration sensation, dysphagia, muscle weakness, muscle atrophy, and muscle cramps; intermediate (3–4 years) for urinary dysfunction and oculomotor dysfunction; and long (6 years) for spasticity (Fig. 2). It was not quantifiable for dystonia, hyperreflexia, and cognitive impairment given that fewer than half of the cohort developed these symptoms over the follow‐up duration (Fig. 2).

### Sample Size Calculation

The modified INAS sum yielded a sample size requirement of 618 participants while the modified INAS count yielded a sample size requirement of 1039 participants. This represents the number of participants required if the modified INAS was used as the primary outcome in a clinical trial.

### External Responsiveness

At baseline, the modified INAS count, sum, and responsive sum showed strong correlations with both the ADL and SARA scales (Pearson correlation coefficients: INAS count, 0.73 and 0.75; modified INAS sum, 0.83 and 0.83; modified INAS responsive sum, 0.81 and 0.80, respectively). However, over a follow‐up period of 2–5 years, correlations weakened significantly for all three modified INAS scores, ranging from 0.15 to 0.32 (data not shown). This is likely due to missing data and may not reflect the true longitudinal external responsiveness; therefore, a mixed‐model regression was undertaken. Here, a 1‐unit change in the normalized modified INAS count (0–1 scale) was associated with a 10.0‐unit change in the ADL scale (SE ± 0.50, 95% CI 9.0, 11.0; *P* < 0.001), and a 9.0‐unit change in the SARA score (SE ± 0.50, 95% CI 8.1, 10.0; *P* < 0.001). A 1‐unit change in the normalized modified INAS sum resulted in a 23.7‐unit change in the ADL scale (SE ± 0.85, 95% CI 22.0, 25.4; *P* < 0.001) and a 20.3‐unit change in the SARA score (SE ± 0.83, 95% CI 18.7, 21.9; *P* < 0.001).

### Factorial Structure and Item Correlations

**FIG. 3:**
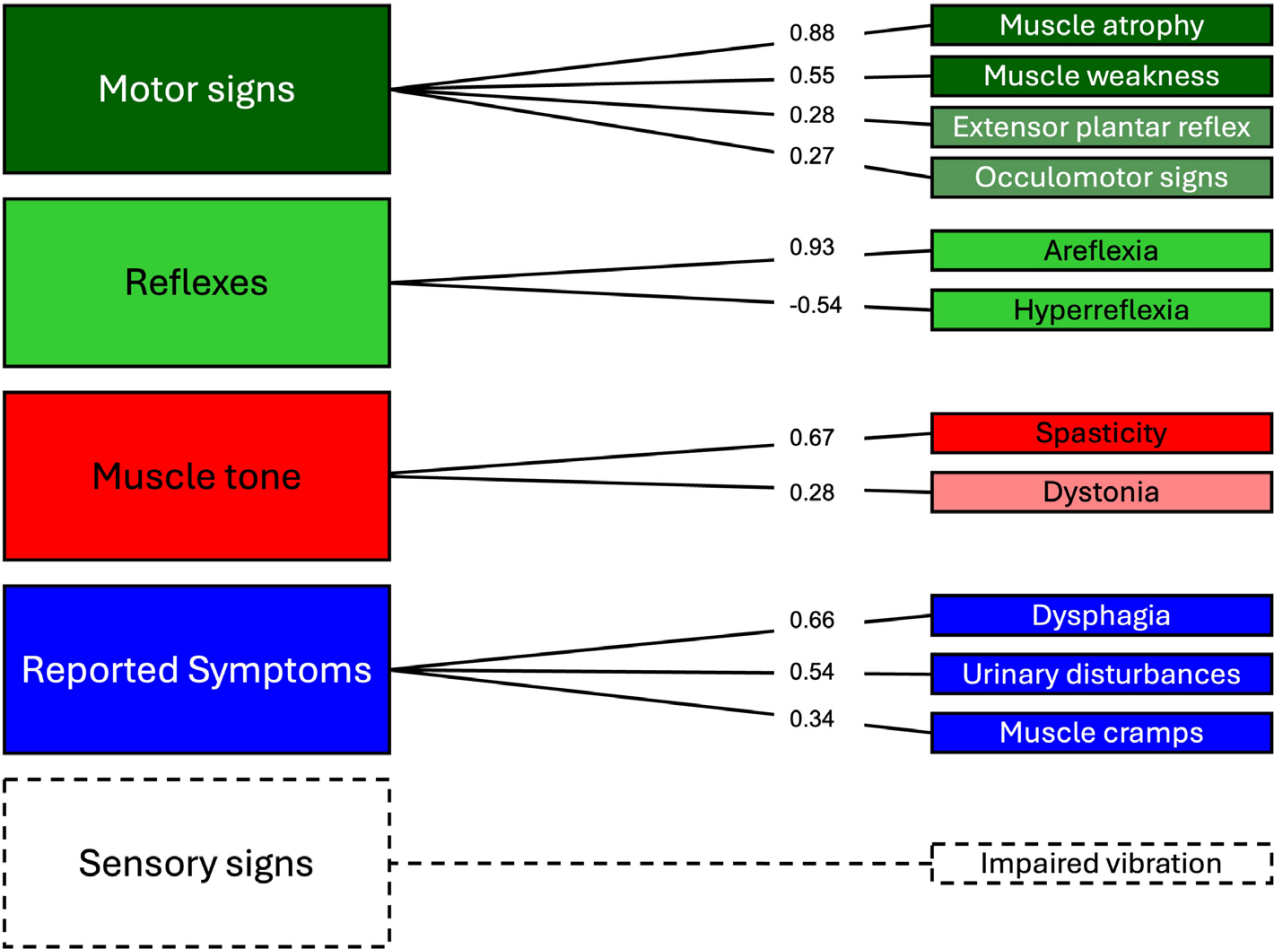
Factorial structure of the modified Inventory of Non‐Ataxia Signs (INAS). Exploratory factor analysis of the modified INAS. Items with factor loadings ≥0.25 are shown (cognitive impairment was excluded). Items with loadings ≥0.3 are displayed in bold colours and those with factor loadings 0.25–0.3 in muted colours. The four‐factor model yielded the most meaningful solution. Here, impaired vibration sensation had a weak loading (0.26) on the Reported Symptoms factor; however, due to its distinct pathophysiological nature and longitudinal responsiveness, it is displayed here as an additional factor in dotted lines. [Color figure can be viewed at wileyonlinelibrary.com]

A four‐factor model yielded the most meaningful interpretation of the modified INAS. The resulting factors were interpreted as reflecting motor signs, reflex abnormalities, muscle tone disturbances, and reported symptoms. Impaired vibration sensation, although only weakly loading onto the reported symptoms factor, were retained due to their distinct clinical relevance and longitudinal responsiveness and displayed as a single factor (Fig. 3). A factor analysis of the original INAS yielded low communalities for the excluded items (<0.14). Item‐to‐item and item‐total correlations are shown in Figures S7 and S9 and Tables S8 and S10, respectively.

## Discussion

In this large, prospective, European, multicenter cohort study, we examined the prevalence and progression of non‐ataxia symptoms and determined the responsiveness of the modified INAS in 1129 patients with genetically confirmed Friedreich's ataxia over 12 years. The non‐ataxia symptoms with consistent worsening over time included muscle weakness, muscle atrophy, impaired vibration sensation, urinary dysfunction, dysphagia, and spasticity. The modified INAS sum demonstrated poor to moderate internal responsiveness over 1–3 years follow‐up but high internal responsiveness over 5 years. In contrast, the modified INAS count exhibited poor responsiveness over 5 years and moderate responsiveness over 10 years. Both scores were strongly correlated with the SARA and ADL scales, established measures of disease severity and functional impairment.

At baseline, spasticity was observed in 28% of patients, muscle weakness in 56%, urinary dysfunction in 38%, and dysphagia in 60%. Prevalence estimates in the literature vary widely, likely reflecting differences in disease duration, age at onset, and methods of assessment. Previous studies reported spasticity in 12%–40%,^20^, ^21^, ^22^ urinary dysfunction in 23%–82%,^23^, ^24^, ^25^, ^26^, ^27^ and dysphagia in 64%^28^ based on clinical evaluation, while standardized assessments identified spasticity and muscle weakness in almost all participants.^29^, ^30^ The multitude of non‐ataxia symptoms highlights the diverse number of anatomical structures affected in Friedreich's ataxia, demonstrating its multisystem nature. Beyond impairments in the proprioceptive pathways, spinal cord, and cerebellum – leading to a characteristic mixed sensory and cerebellar ataxia – damage to the corticospinal tracts contributes to spasticity, urinary disturbances, and muscle weakness. Additionally, cranial nerve nuclei impairments likely cause dysphagia, while skeletal muscle involvement leads to weakness and atrophy.^31^ Furthermore, emerging evidence suggests that dysfunction of the cortico‐reticular and reticulo‐spinal pathways also play a role in the development of spasticity.^32^, ^33^, ^34^

The modified INAS sum showed high responsiveness in the medium and long term but not in the short term; in contrast, the modified INAS count exhibited poor responsiveness in the medium term and moderate responsiveness in the long term. Our findings align with the INAS validation study, with a similar reported SRM of the INAS count (0.31 over 2 years compared with 0.27 in our cohort).^13^ By comparison, recent analyses of the EFACTS cohort highlighted the superior responsiveness of other measures such as the SARA (SRM 0.54 over 2 years) and the ADL scale (SRM 0.67 over 2 years) in the total cohort.^1^, ^15^ Conversely, in line with our initial hypothesis, the modified INAS sum outperformed the SARA in non‐ambulatory patients (SRM 0.47 vs. 0.39 at 3 years), although the ADL scale remained superior (SRM 0.82 at 3 years).^1^ The modified INAS sum demonstrated comparable short‐term responsiveness to the total cohort (SRM 0.47 vs. 0.53 at 3 years) and slightly higher medium‐term responsiveness (SRM 0.82 vs. 0.80 at 5 years). Conversely, the modified INAS count exhibited ceiling effects in this population, with minimal or negative SRM values. Interestingly, the modified INAS sum performed very well in children, compared with adults, reaching high responsiveness (SRM 1.07) over 3 years, which may be due to the faster disease progression linked to longer GAA repeat length in this group.

The relatively modest responsiveness of the modified INAS in the total cohort in the short term underscores the challenges of developing effective outcome measures for Friedreich's ataxia, given its slow progression and substantial heterogeneity.^1^, ^15^, ^35^ Compared with the linear progression of ataxia, non‐ataxia signs and symptoms appear to progress at a more variable and slower rate.

The most responsive modified INAS items were muscle weakness, muscle atrophy, impaired vibration sensation, urinary dysfunction, and dysphagia. Spasticity and dystonia were only responsive in the medium and long term, with dystonia showing somewhat inconsistent responsiveness likely due to sampling variability, while reflex‐related items (areflexia, hyperreflexia, extensor plantar reflex) and cognitive impairment demonstrated poor responsiveness. The limited responsiveness of reflex status is unsurprising, as areflexia is typically present at diagnosis,^36^ and hyperreflexia is relatively rare.^37^, ^38^ High inter‐rater variability may also explain the unsuitability of these items as progression markers. Prior research suggests substantial variability in assessing tone and reflexes compared with other neurological signs.^39^, ^40^ To improve responsiveness, aligning the INAS with extensively validated scales for specific items may be beneficial, such as the Tardieu scale for spasticity,^41^ the Medical Research Council Scale for muscle weakness,^42^ the Actionable Bladder Symptom Screening Tool (ABSST) for urinary dysfunction,^43^ and the Swallowing Quality of Life Questionnaire (SWAL‐QOL) for dysphagia.^44^, ^45^

We also explored a modified INAS responsive sum that included only the most responsive items. This approach offered a slight improvement in short‐term responsiveness (1–2 years) but no significant advantage in the medium and long term. Given the post‐hoc nature of this analysis, these findings should be interpreted cautiously.

### Strengths and Weaknesses

The strengths of this study include its large sample size and very long follow‐up duration of up to 12 years, which provide robust insights into the longitudinal progression of non‐ataxia symptoms in patients with Friedreich's ataxia. Notwithstanding these strengths, several limitations must be acknowledged. First, the quantity of missing data in the modified INAS count and decrease in sample size over time present challenges, potentially introducing bias and reducing the generalizability of the findings, particularly as missing values were more frequent in patients with more severe impairment. The pattern of missing data can be classified as intermittent such that data may be missing at one visit but are available again at subsequent visits and are likely categorizable as missing at random, given that missingness appeared to depend on observed data. Our analysis using mixed‐effects models utilizes maximum likelihood estimation and incorporates all available data, leveraging the observed repeated measure data without requiring multiple imputation.^46^ Similarly, we performed time‐to‐event analysis, which handles loss to follow‐up through censoring and post‐event missing data.^46^ Additionally, while the modified INAS scale captures a wide range of symptoms, it relies on subjective assessments that may be influenced by inter‐rater variability. Future research could consider aligning modified INAS items with validated rating scales to improve reliability. Misinterpretation of certain items, such as spasticity of gait, further complicates scoring. However, sensitivity analysis demonstrated that its exclusion did not affect the results.

Symptom fluctuations over time present another limitation, as the INAS records symptoms at discrete time points, which may not fully capture short‐term variations or changes due to environmental or physiological factors. Finally, the absence of standardized protocols for symptom evaluation and data collection across centers likely contributes to variability, potentially impacting the precision of the findings.

## Conclusions

Our results emphasize the high prevalence and progressive worsening of many neurological non‐ataxia symptoms in patients with Friedreich's ataxia and attendant morbidity, underscoring the complex multisystem nature of the disease. While the modified INAS is not suitable as a primary outcome measure in clinical trials due to its limited responsiveness in the short term, it may be valuable as a secondary outcome measure supplementing the SARA and ADL scales and contributing to a more holistic assessment of neurological features. In particular, the modified INAS may provide a valuable assessment tool in non‐ambulatory patients, where ataxia‐focused measures may be constrained by ceiling effects, and in children who typically progress faster. The added value of the continuous scoring of the modified INAS sum is its greater granularity, capturing both the onset of new symptoms and the progression of existing ones.

## Author Roles

(1) Research Project: A. Study Concept, B. Data Acquisition, C. Supervision; (2) Statistical Analysis: A. Design of Current Analysis, B. Analysis and Interpretation of Data; (3) Manuscript Preparation: A. Writing of the First Draft, B. Review and Revision; (4) Principal Investigator (PI) of EFACTS.

S.A.L.: 1A, 2A, 2B, 3A, 3B.

I.D.: 1A, 2A, 2B, 3B.

P.G.: 1B, 1C, 3B, 4A.

M.H.P.: 1B, 3B.

C.M.: 1B, 1C, 3B, 4A.

A.D.: 1B, 1C, 3B, 4A.

C.E.: 1B, 1C, 3B.

S.B.: 1B, 1C, 3B, 4A.

W.N.: 1B, 1C, 3B.

T.Klopstock: 1B, 1C, 3B, 4A.

C.S.: 1B, 1C, 3B.

F.J.R.d.R.G.: 1B, 1C, 3B, 4A.

L.S.: 1B, 1C, 3B, 4A.

Z.F.: 1B, 1C, 3B..

T.Klockgether: 1B, 1C, 3B.

M.G.‐E.: 1B, 1C, 3B.

I.G.: 1B, 1C, 3B.

M.R.: 1B, 1C, 3B.

M.P.: 1B, 1C, 3B, 4A.

H.J.: 1B, 1C, 3B.

R.D.H.: 1B, 1C, 3B.

J.B.S.: 1A, 1B, 1C, 2A, 3B, 4A.

K.R.: 1A, 1B, 1C, 2A, 3B.

EFACTS Study Group: 1B, 1C, 3B.

All authors gave final approval of the version to be published and take responsibility for the conduct of the research.

## Supporting information


**Data S1.** Supporting Information

## Appendix A EFACTS Study Group

Austria: Elisabetta Indelicato, Matthias Amprosi (Medical University Innsbruck). Italy: Cinzia Gellera, Alessia Mongelli, Anna Castaldo, Mario Fichera, (Fondazione IRCCS Istituto Neurologico Carlo Besta, Milan); Enrico Bertini, Gessica Vasco (IRCCS Bambino Gesù Children's Hospital, Rome, Italy). France: Marie Biet, Marie Lorraine Monin (Pitié‐Salpêtrière University Hospital, Paris). Germany: Florian Holtbernd, Nikolina Brcina, Christian Hohenfeld (RWTH Aachen University, Aachen); Florentine Radelfahr, Almut T. Bischoff (University of Munich, Munich); Stefanie N. Hayer (University of Tübingen, Tübingen). Greece: Georgios Koutsis, Marianthi Breza (National and Kapodistrian University of Athens, Eginitio Hospital, Athens, Greece). Spain: Francesc Palau, Mar O'Callaghan (Sant Joan de Déu Children's Hospital and Research Institute, and CIBERER, Barcelona, Spain). UK: Gilbert Thomas‐Black, Katarina Manso, Nita Solanky, Robyn Labrum (Queen Square Institute of Neurology, London).
