# Supplementary material for: Analysis of a Modified Version of the Inventory of Non‐Ataxia Signs Over 12 Years in Patients with Friedreich's Ataxia in the EFACTS Study
Source: Mov Disord. 2025 Oct 10;41(1):200–11. doi: 10.1002/mds.70084 (PMC12882042; doi:10.1002/mds.70084)
Supplement: Supplementary file 1 — Data S1. Supporting Information [file MDS-41-200-s001.docx]

**Supplementary material: Analysis of a modified version of the inventory of non-ataxia symptoms over 12 years in patients with Friedreich’s ataxia in the EFACTS study**

**Table S1**. Items of the INAS and scoring of the INAS summed score

| Source | Item | Components | Point range | |
| --- | --- | --- | --- | --- |
| Examination | Areflexia | Biceps tendon reflex | 0 | 3 |
|  |  | Patellar tendon reflex |  |  |
|  |  | Achilles tendon reflex |  |  |
|  | Extensor plantar response | Unilateral vs bilateral | 0 | 2 |
|  | Paresis | Face/tongue | 0 | 15 |
|  |  | Upper limbs proximal |  |  |
|  |  | Upper limbs distal |  |  |
|  |  | Lower limbs proximal |  |  |
|  |  | Lower limbs distal |  |  |
|  | Spasticity | Upper limbs | 0 | 6 |
|  |  | Lower limbs |  |  |
|  | Muscle atrophy | Face/tongue | 0 | 15 |
|  |  | Upper limbs proximal |  |  |
|  |  | Upper limbs distal |  |  |
|  |  | Lower limbs proximal |  |  |
|  |  | Lower limbs distal |  |  |
|  | Dystonia | Face/tongue | 0 | 15 |
|  |  | Neck |  |  |
|  |  | Trunk |  |  |
|  |  | Upper limbs |  |  |
|  |  | Lower limbs |  |  |
|  | Impaired vibration sensation | Left foot | 0 | 6 |
|  |  | Right foot |  |  |
|  | Oculomotor dysfunction | Smooth pursuit | 0 | 10 |
|  |  | Square wave jerks |  |  |
|  |  | Downbeat-nystagmus |  |  |
|  |  | Gaze-evoked nystagmus horizontal |  |  |
|  |  | Gaze-evoked nystagmus vertical |  |  |
|  |  | Ophthalmoparesis horizontal |  |  |
|  |  | Ophthalmoparesis vertical |  |  |
|  |  | Slowing of saccades |  |  |
|  |  | Hypometric saccades |  |  |
|  |  | Hypermetric saccades |  |  |
| History | Muscle cramps |  | 0 | 3 |
|  | Urinary dysfunction |  | 0 | 3 |
|  | Dysphagia |  | 0 | 3 |
|  | Cognitive impairment |  | 0 | 3 |
| Total score | | |  | 84 |

**Table S2**. Items where modifications were considered for developing the modified INAS

| **Item** | **Prevalence (n, %)** | **Literature search strategy** | **Literature findings** | **Outcome** |
| --- | --- | --- | --- | --- |
| Resting tremor | 18 (1.6%) | ("resting tremor"[All Fields] OR "rest tremor"[All Fields]) | No studies identified | excluded |
| Chorea | 19 (1.7%) | ("chorea"[MeSH Terms] OR "chorea"[All Fields] OR "choreas"[All Fields] OR "choreiform"[All Fields] OR ("choreic"[All Fields] OR "choreics"[All Fields])) | 103 search results including 3 case reports on four patients, of whom two were compound heterozygous ^1-3^ | excluded |
| Rigidity | 28 (2.5%) | ("muscle rigidity"[MeSH Terms] OR ("muscle"[All Fields] AND "rigidity"[All Fields]) OR "muscle rigidity"[All Fields] OR "rigid"[All Fields] OR "rigidity"[All Fields] OR "rigidities"[All Fields] OR "rigidness"[All Fields]) | Eight search results of which none were relevant | excluded |
| Myoclonus | 43 (3.9%) | ("myoclonus"[MeSH Terms] OR "myoclonus"[All Fields] OR ("myoclonal"[All Fields] OR "myoclonic"[All Fields])) | 72 search results including two case reports ow which one was compound heterozygous^1 4^ | excluded |
| Fasciculations | 44 (4.0%) | "fasciculation*"[All Fields] | Two results which were not relevant | excluded |
| Dystonia | 50 (4.5%) | ("dystonia"[MeSH Terms] OR "dystonia"[All Fields] OR "dystonias"[All Fields] OR "dystonic disorders"[MeSH Terms] OR ("dystonic"[All Fields] AND "disorders"[All Fields]) OR "dystonic disorders"[All Fields] OR ("dystonic"[All Fields] OR "dystonics"[All Fields])) | 50 search results including one case report^5^  See manuscript for additional evidence supporting inclusion of this item. | retained |
| Dysphagia | 628 (57%) | ("deglutition disorders"[MeSH Terms] OR ("deglutition"[All Fields] AND "disorders"[All Fields]) OR "deglutition disorders"[All Fields] OR "dysphagia"[All Fields] OR "dysphagias"[All Fields] OR ("deglutition"[MeSH Terms] OR "deglutition"[All Fields] OR "swallowed"[All Fields] OR "swallowing"[All Fields] OR "swallowings"[All Fields] OR "swallowable"[All Fields] OR "swallower"[All Fields] OR "swallowers"[All Fields] OR "swallows"[MeSH Terms] OR "swallows"[All Fields] OR "swallow"[All Fields]) OR ("deglutition"[MeSH Terms] OR "deglutition"[All Fields] OR "deglutitions"[All Fields] OR "deglutitive"[All Fields])) | 17 search results including three cohort studies including a longitudinal study identifying deterioration in swallowing function over 12 months^6-8^ | added |
| Muscle cramps or muscle spasms | 476 (53) | ((("muscle s"[All Fields] OR "muscles"[MeSH Terms] OR "muscles"[All Fields] OR "muscle"[All Fields]) AND "spasm*"[All Fields]) OR (("muscle s"[All Fields] OR "muscles"[MeSH Terms] OR "muscles"[All Fields] OR "muscle"[All Fields]) AND "cramp*"[All Fields])) | 7 results with one case report of baclofen treatment for severe spasms^9^  Muscle spasms/cramps mentioned in international guidelines^10^ | added |

*Legend: the search strategy for each symptom was combined with AND with a search strategy for Friedreich's ataxia as follows: AND "ataxia"[All Fields]) OR "Friedreich ataxia"[All Fields] OR "frda"[All Fields]) OR "Friedreich ataxia"[MeSH Major Topic]). The search was performed in MEDLINE.*

*References:*

1. Zhu D, Burke C, Leslie A, et al. Friedreich's ataxia with chorea and myoclonus caused by a compound heterozygosity for a novel deletion and the trinucleotide GAA expansion. *Movement Disorders* 2002;17(3):585-89. doi: <https://doi.org/10.1002/mds.10175>

2. Hanna MG, Davis MB, Sweeney MG, et al. Generalized chorea in two patients harboring the Friedreich's ataxia gene trinucleotide repeat expansion. *Mov Disord* 1998;13(2):339-40. doi: 10.1002/mds.870130223

3. Spacey SD, Szczygielski BI, Young SP, et al. Malaysian siblings with friedreich ataxia and chorea: a novel deletion in the frataxin gene. *Can J Neurol Sci* 2004;31(3):383-6. doi: 10.1017/s0317167100003498

4. Jain RS, Kumar S, Tejwani S. Familial segmental spinal myoclonus: a rare clinical feature of Friedreich's ataxia. *Springerplus* 2015;4:330. doi: 10.1186/s40064-015-1121-5 [published Online First: 20150708]

5. Rota S, Marchina E, Todeschini A, et al. Very late-onset friedreich ataxia with laryngeal dystonia. *Case Rep Neurol* 2014;6(3):287-90. doi: 10.1159/000370062 [published Online First: 20141212]

6. Keage MJ, Delatycki MB, Gupta I, et al. Dysphagia in Friedreich Ataxia. *Dysphagia* 2017;32(5):626-35. doi: 10.1007/s00455-017-9804-4 [published Online First: 20170504]

7. Keage M, Delatycki MB, Dyer J, et al. Changes detected in swallowing function in Friedreich ataxia over 12 months. *Neuromuscul Disord* 2019;29(10):786-93. doi: 10.1016/j.nmd.2019.08.013 [published Online First: 20190906]

8. Vogel AP, Brown SE, Folker JE, et al. Dysphagia and swallowing-related quality of life in Friedreich ataxia. *J Neurol* 2014;261(2):392-9. doi: 10.1007/s00415-013-7208-4 [published Online First: 20131227]

9. Kalyvas AV, Drosos E, Korfias S, et al. Intrathecal Baclofen Therapy for Painful Muscle Spasms in a Patient with Friedreich's Ataxia. *Stereotact Funct Neurosurg* 2018;96(2):127-30. doi: 10.1159/000489220 [published Online First: 20180608]

10. Corben LA, Collins V, Milne S, et al. Clinical management guidelines for Friedreich ataxia: best practice in rare diseases. *Orphanet Journal of Rare Diseases* 2022;17(1):415. doi: 10.1186/s13023-022-02568-3

**Table S3**. Results of univariate regression for demographic and clinical factors original and INAS sum score at baseline

|  | **INAS count** | | | **INAS sum score** | | |
| --- | --- | --- | --- | --- | --- | --- |
| **Characteristic** | **Beta** | **95% CI** | **p-value** | **Beta** | **95% CI** | **p-value** |
| Sex (female) | 0.18 | -0.10, 0.46 | 0.2 | 1.1 | -0.18, 2.3 | 0.094 |
| Age at symptom onset | -0.04 | -0.05, -0.03 | <0.001 | -0.23 | -0.28, -0.17 | <0.001 |
| Disease duration | 0.1 | 0.09, 0.11 | <0.001 | 0.56 | 0.51, 0.60 | <0.001 |
| GAA repeats shorter allele | 0 | 0.00, 0.00 | <0.001 | 0.01 | 0.01, 0.01 | <0.001 |
| ADL total score | 0.2 | 0.19, 0.21 | <0.001 | 1 | 0.97, 1.1 | <0.001 |
| SARA total score | 0.16 | 0.15, 0.16 | <0.001 | 0.78 | 0.75, 0.82 | <0.001 |
| Cardiac hypertrophy | 0.66 | 0.37, 0.96 | <0.001 | 2.7 | 1.4, 4.0 | <0.001 |

*Legend: Univariate linear regression with listed characteristics as respective independent variables and original and INAS sum as the dependant variables. Abbreviations: ADL: activities of daily living; CI: confidence interval; GAA: guanine adenine adenine; SARA scale for the assessment and rating of ataxia.*

**Table S4**. Number of patients followed up per visit

| **Visit** | **Sample size** |
| --- | --- |
| 1 | 1129 |
| 2 | 832 |
| 3 | 732 |
| 4 | 544 |
| 5 | 523 |
| 6 | 454 |
| 7 | 380 |
| 8 | 294 |
| 9 | 186 |
| 10 | 161 |
| 11 | 157 |
| 12 | 175 |

**Table S5**. Missingness of items in original INAS count

| **visit** | **hyperreflexia** | **areflexia** | **extensor plantar reflex** | **spasticity** | **muscle weakness** | **muscle atrophy** | **fasciculations** | **myoclonus** | **rigidity** | **chorea** | **dystonia** | **resting tremor** | **impaired vibration** | **occulomotor dysfunction** | **urinary dysfunction** | **cognitive impairment** | **INAS count** |
| --- | --- | --- | --- | --- | --- | --- | --- | --- | --- | --- | --- | --- | --- | --- | --- | --- | --- |
| **1** | 2.7 | 2.7 | 3.7 | 3.3 | 3.3 | 3 | 2.6 | 2.5 | 2.6 | 2.4 | 2.5 | 2.2 | 5.8 | 8.8 | 2.4 | 2.7 | 16.1 |
| **2** | 3.6 | 3.6 | 5 | 4.6 | 3.7 | 4.2 | 3.5 | 3.6 | 3.8 | 3.5 | 3.5 | 3.4 | 5.6 | 8.9 | 3.1 | 3.6 | 13.7 |
| **3** | 3.6 | 3.6 | 5.1 | 3 | 3.3 | 3.6 | 2.9 | 2.9 | 2.9 | 2.9 | 2.9 | 2.9 | 6 | 7.1 | 2.7 | 3.4 | 12.6 |
| **4** | 9 | 9 | 9.7 | 8.1 | 7.7 | 7.9 | 7.5 | 7.7 | 8.3 | 7.7 | 7.9 | 7.7 | 13.1 | 11.8 | 7.7 | 7.4 | 19.7 |
| **5** | 14.7 | 14.7 | 16.3 | 15.9 | 15.3 | 14.5 | 14.9 | 14.9 | 14.5 | 14.7 | 14.9 | 14.7 | 18.2 | 17.2 | 14.1 | 15.1 | 23.9 |
| **6** | 6.6 | 6.6 | 12.8 | 7 | 7.5 | 7.7 | 5.9 | 5.9 | 5.9 | 5.9 | 6.2 | 6.6 | 11.5 | 8.8 | 6.8 | 7.7 | 20.5 |
| **7** | 7.9 | 7.9 | 9.7 | 7.9 | 8.9 | 8.4 | 7.1 | 7.6 | 7.4 | 7.6 | 7.6 | 7.4 | 12.1 | 9.2 | 7.9 | 8.7 | 18.7 |
| **8** | 7.5 | 7.5 | 8.2 | 6.8 | 7.5 | 7.8 | 5.8 | 5.8 | 6.1 | 6.1 | 6.1 | 6.1 | 9.5 | 10.9 | 6.8 | 8.2 | 18.7 |
| **9** | 17.2 | 17.2 | 18.8 | 17.7 | 17.2 | 17.7 | 17.2 | 17.2 | 17.2 | 17.2 | 17.7 | 17.2 | 21 | 18.8 | 18.3 | 18.8 | 25.3 |
| **10** | 31.7 | 31.7 | 32.3 | 31.7 | 32.3 | 31.7 | 32.3 | 31.7 | 31.7 | 31.7 | 31.7 | 31.7 | 34.8 | 33.5 | 32.3 | 32.3 | 37.3 |
| **11** | 13.4 | 13.4 | 14 | 12.1 | 12.7 | 12.1 | 12.7 | 12.1 | 12.1 | 12.7 | 12.7 | 12.1 | 12.7 | 17.2 | 11.5 | 12.1 | 21.7 |
| **12** | 9.1 | 9.1 | 10.9 | 9.1 | 9.1 | 8.6 | 8.6 | 8.6 | 9.1 | 8.6 | 8.6 | 8.6 | 10.3 | 12 | 8.6 | 8.6 | 17.7 |

**Table S6**. Missingness of items in the modified INAS

| **visit** | **dysphagia** | **muscle cramps** | **modified INAS sum** | **modified INAS responsive sum** |
| --- | --- | --- | --- | --- |
| **1** | 2.3 | 2.9 | 16.4 | 8.7 |
| **2** | 3.2 | 3.7 | 13.7 | 7.6 |
| **3** | 2.9 | 3.1 | 12.7 | 7.7 |
| **4** | 7.4 | 7.5 | 19.5 | 14.3 |
| **5** | 14.5 | 14.7 | 24.1 | 20.5 |
| **6** | 6.8 | 7.7 | 20.5 | 14.1 |
| **7** | 7.6 | 8.9 | 18.9 | 15 |
| **8** | 7.1 | 8.2 | 19.4 | 13.9 |
| **9** | 17.7 | 17.7 | 25.3 | 22.6 |
| **10** | 32.3 | 32.3 | 37.3 | 36 |
| **11** | 11.5 | 12.1 | 21 | 14 |
| **12** | 8 | 9.1 | 17.1 | 12 |

**Figure S7**. Item-to-item correlations for the modified INAS


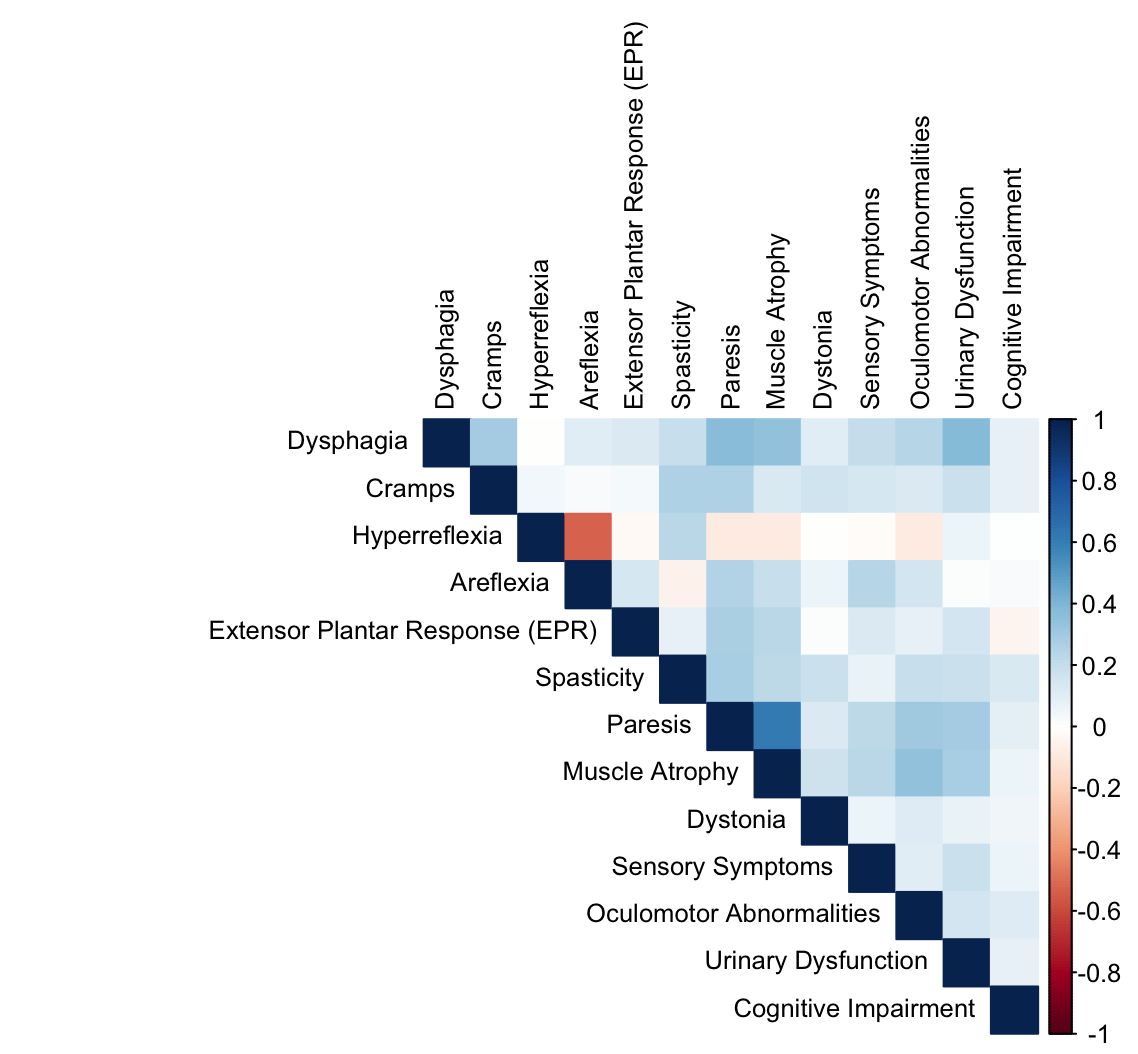


**Table S8.** Item-total correlations for the modified INAS

| **Item** | **item-total correlation** |
| --- | --- |
| Dysphagia | 0.52 |
| Muscle cramps | 0.308 |
| Hyperreflexia | -0.105 |
| Areflexia | 0.26 |
| Extensor plantar reflex | 0.272 |
| Spasticity | 0.377 |
| Muscle weakness | 0.66 |
| Muscle atrophy | 0.639 |
| Dystonia | 0.242 |
| Impaired vibration sensation | 0.345 |
| Occulomotor signs | 0.424 |
| Urinary dysfunction | 0.402 |
| Cognitive impairment | 0.189 |

**Figure S9**. Item-to-item correlations for the original INAS


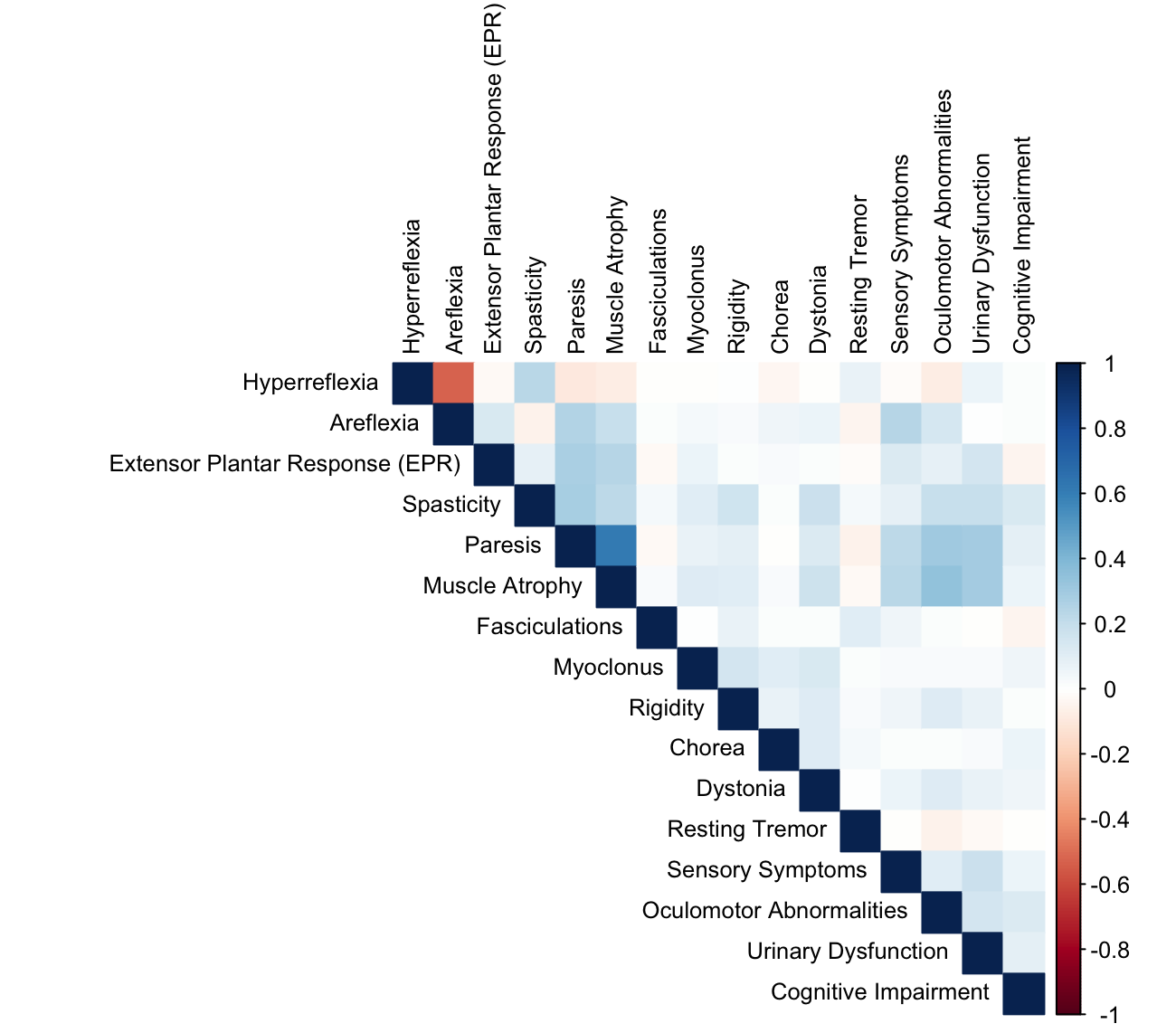


**Table S10.** Item-total correlations for the modified INAS

| **Item** | **Item-total correlation** |
| --- | --- |
| Hyperreflexia | -0.076 |
| Areflexia | 0.15 |
| Extensor plantar reflex | 0.246 |
| Spasticity | 0.345 |
| Muscle weakness | 0.57 |
| Muscle atrophy | 0.569 |
| Fasciculations | 0.023 |
| Myoclonus | 0.142 |
| Rigidity | 0.18 |
| Chorea | 0.062 |
| Dystonia | 0.213 |
| Resting tremor | -0.014 |
| Impaired vibration sensation | 0.29 |
| Occulomotor signs | 0.339 |
| Urinary dysfunction | 0.339 |
| Cognitive impairment | 0.13 |
